# The application of plotted surgical guides for pilot-guided and full-guided implant insertion– a prospective model study in a cohort of undergraduate dental students

**DOI:** 10.1186/s40729-025-00642-6

**Published:** 2025-08-16

**Authors:** Rudolf Reil, Michael Krimmel, Christina Weismann, Ali-Reza Ketabi, Andreas Naros, Siegmar Reinert, Matthias C. Schulz

**Affiliations:** 1 Department of Oral and Maxillofacial Surgery (Head: Prof. Dr. Dr. Bernd Lethaus), University Hospital Tübingen, Eberhard Karls Universität Tübingen, Osianderstr. 2-8, D-72076 Tübingen, Germany; 2 Department of Orthodontics (Head: Prof. Dr. Bernd Koos), University Hospital Tübingen, Eberhard Karls Universität Tübingen, Osianderstr. 2-8, D-72076 Tübingen, Germany; 3 Department of Prosthodontics (Head: Prof. Dr. Andree Piwowarczyk), School of Dentistry, Faculty of Health, Witten/Herdecke University, Alfred-Herrhausen-Str. 45, D-58455 Witten, Germany; 4 Private Dental Office, Kirchheimer Straße 71, D-70619 Stuttgart-Sillenbuch, Germany

**Keywords:** Full-guided implant insertion, Laboratory study, Orientation template, Three-dimensional plotting

## Abstract

**Purpose:**

Implant dentistry is an established therapy option with sufficient long-term success for the replacement of missing teeth. Education in implant dentistry should not only focus on theory but also on practical courses. The purpose of the current examination was to assess the accuracy of fully guided and pilot-drill guided implant insertion applying plotted static guides in a cohort of undergraduate dental students.

**Methods:**

Matching a three-dimensional set of radiographic data and surface scans of 51 artificial mandibular models, 51 surgical templates were produced by plotting. Metal sleeves allowing either a pilot-drill or fully guided implant insertion were inserted alternatively in region 36 and 46. A total of 102 implants were inserted by 51 undergraduates. Subsequently, the positions of the implants were analyzed radiographically considering the accuracy. Additionally, the time required for implant insertion was recorded and a questionnaire was completed. Statistical analysis followed.

**Results:**

In general, the accuracy of fully guided implant insertion was higher compared to pilot-drill guided. Mean three-dimensional deviation was 2.24 ± 0.38 degrees for fully guided vs. 4.51 ± 2.20 degrees for pilot-drill guided implant insertion. Time required for fully guided implant insertion was statistically significant higher compared to pilot-drill guided (15:22 ± 5:22 vs. 9:35 ± 3:58 min, *p* < 0.01). The returned questionnaires reported a high interest but a self-assessed minor previous knowledge in implant dentistry.

**Conclusion:**

The examination could show that inexperienced undergraduates benefited from fully guided implant insertion in a laboratory set-up. Based on the questionnaires there is a distinct demand for an extended education in implant dentistry among undergraduate students.

**Graphical abstract:**

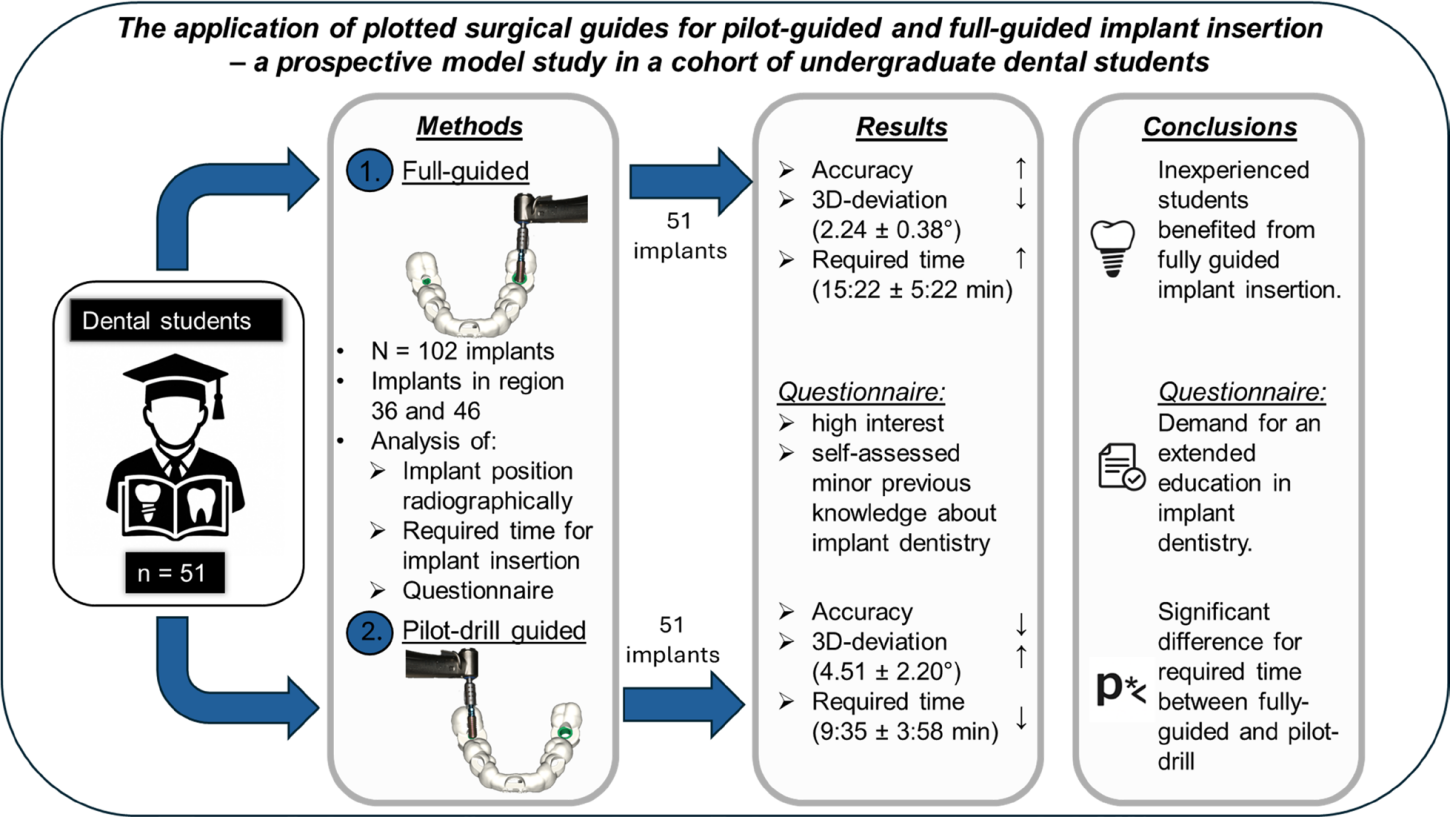

## Background

In recent years, implant dentistry has developed to an established treatment option for partially and fully edentulous jaws. The implant position should be planned according to anatomical and prosthetic aspects to allow an ideal position and to avoid the damage of adjacent anatomical structures [1]. Currently, there is a variety of methods available to transfer the planned implant position into the clinical situation, ranging from free-hand implant insertion to pilot-drill guided and full-guided implant insertion as static methods [2]. Free-hand implant insertion is also referred as brain-guided implant insertion by determining the implant position and angulation according to the clinical situation without the use of a surgical guide [2]. The pilot-drill guide is solely applied for drilling the first implant cavity determining the implant position and angulation based on three-dimensional planning. The subsequent preparation of the implant cavity is performed free-hand allowing minor correction of the angulation [2]. The application of full-guided implant insertion requires three-dimensional planning using computed tomography (CT) or cone beam computed tomography (CBCT) scans. The surgical guide is used for every single step of the implant cavity preparation from the pilot-drill to the implant insertion [2]. By using the fully guided implant insertion only minor deviations are possible. Considering the transfer accuracy of the planned implant position, the mentioned methods show differences: A higher accuracy was achieved when a fully guided mode was used compared to pilot-drill guided or free-hand implant insertion in previous studies [3–9]. On the other hand, these methods diverge in planning and manufacturing efforts and thus, in the expense. In the recent years, the use of three-dimensionally manufactured templates has increased [10]. With the increasing spread of digital implant planning software, optical scanners and cost-effective three-dimensional printers, it is possible to individually design and manufacture the templates in house [10, 11]. Thus, a simple and brief process in manufacturing surgical templates for implant insertion seems possible even in daily practice. Furthermore, education of unexperienced surgeons is possible with the use of guided surgery as supervision of the single steps is possible [12]. Additionally, it could be shown in recent studies, that both, experienced and unexperienced surgeons benefit from guided surgery [5, 13, 14].

Considering the undergraduate education in implant dentistry, the demand for the participation in training is increasing [15–18]. While in several universities basic planning with insertion of implants in model situations is done, other universities even offer implant placement in patients [9, 14, 19]. There are studies suggesting that education in implant dentistry should be extended [20, 21]. Especially guided implant insertion would allow unexperienced students safe and reproducible results with avoidance of incorrect implant positions and injury of important anatomical structures [22]. Furthermore, the standardized analysis of the accuracy enables the instructor to give direct feedback [23].

The aim of the present examination was to evaluate the accuracy of transferring the planned implant position into an artificial mandible using three-dimensional plotted static guides allowing full-guided and pilot-drill guided implant insertion in a cohort of undergraduates. We hypothesized that fully guided implant insertion leads to a higher accuracy compared to pilot-drill guided. It was expected that the full-guided method required more time as a higher number of steps are included in the procedure. Additionally, individual factors, e.g. age, sex, handedness, and a completed education before dental school were analyzed. Furthermore, the undergraduates were asked to complete a questionnaire in order to evaluate education in dental implantology.

## Methods

Prior to the start of the examination, the study protocol was approved by the local ethical review board (file reference: 708/2020/BO2). The study was performed in accordance with the Declaration of Helsinki in its current version [24]. All participants gave their written informed consent before taking part in the hands-on course. A sample calculation was performed based on the main parameter (three-dimensional angle of the implant axis) obtained in the previous study revealing a sample size of 34 participants with a significance level of α = 0.05 and a statistical power of 80% as sufficient [9].

**Fig. 1:**
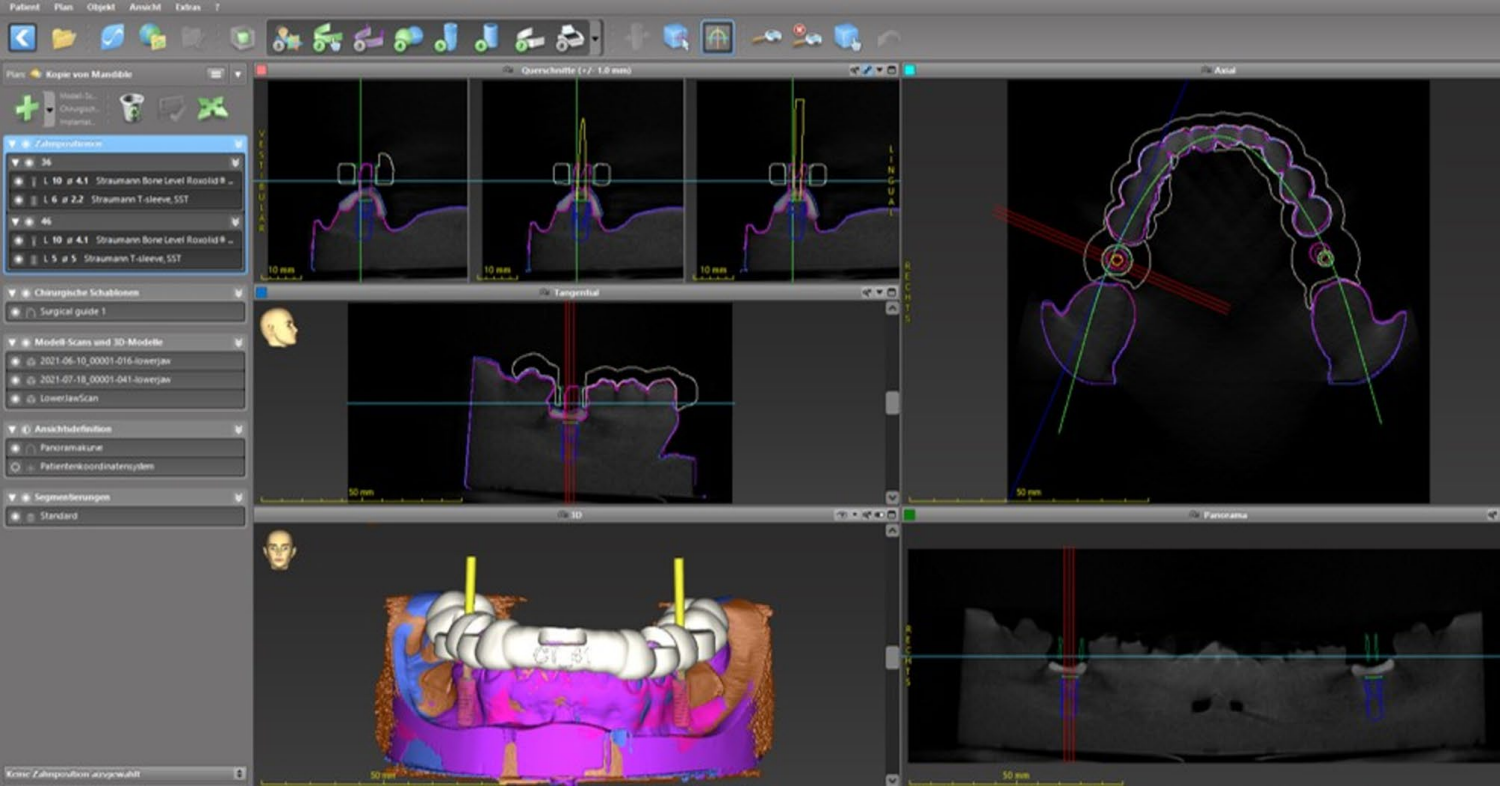
Virtual planning of the implant position and angulation in region 36 and 46 using the coDiagnostiX™ software. Images in clockwise order: Upper right– horizontal plane. Lower right– panoramic view of the mandibular model with virtually inserted implants in region 36 and 46. Lower left– three-dimensional reconstruction of the mandibular model. Center left– Lateral aspect of the virtually inserted implant in region 46. Upper left– coronal aspect of the virtually inserted implant in region 46. White: virtually planned template. Yellow: planned implant axis. Pink: surface scan of the mandibular model. Green line: panoramic plane. Red lines: coronar plane. Blue line: sagittal plane. Light blue line: horizontal plane

A total of 51 artificial mandibular models (Implantec Dentallabor, Amstetten, Germany) with a single-tooth gap in regions 36 and 46 were used for the examination. The models were consecutively numbered starting from 1 to 51. In a first step, a surface scan of the models using a laboratory scanner (Tizian Smart-Scan Plus 2.0, Schütz Dental Group, Rosbach, Germany) with the software TizianCreativ RT– Workflow 1.0, Version 2.4 (exocad GmbH, Darmstadt, Germany) and a cone beam computed tomography (Veraviewepocs 3D, J. Morita Corporation, Osaka, Japan) were performed. For the laboratory scanner, the scan accuracy was set up at ± 8 μm. The data was saved in Standard Tessellation Language (STL). For the CBCT, the following parameters were used: tube voltage: 90 kV; current: 8 mA; exposure time: 9.4 s field of view: 100 × 100 × 50 mm; voxel size: 0.125 × 0.125 × 0.125 mm. The acquired data was saved in Digital Imaging and Documentation in Medicine (DICOM) format. Next, both data sets were transferred into a planning software (coDiagnostiX™ Version 10.2, Dental Wings, Chemnitz, Germany). The planning of the implant positions in region 36 and 46 was performed according to clinical requirements regarding bone width and distance and angulations of the adjacent teeth (Fig. 1). In order to avoid a bias regarding the implant site, pilot-drill guided and fully guided implant insertion were distributed as depicted in Table 1. Based on the implant positions, templates covering the dental arch of the model from tooth 37 to tooth 47 were designed by an experienced dental technician (R.R.) under guidance of an experienced oral surgeon (M. C. S.). Three fenestrations in regions 31/41, 34 and 44 were designed in order to check the fitting accuracy of the templates on the corresponding model. A material thickness of 3 mm with large connectors was chosen. The printing off-set was adjusted to 0.2 mm. Next, the STL data set was transferred to the three-dimensional printer to produce the templates. The production of the templates was carried out in an external dental laboratory (Zahntechnik Reil GmbH, Nabburg, Germany) using the printer 5100 Next Dent (3D Systems GmbH, Moerfelden-Walldorf, Germany) with the software 3D Sprint (Version 3.1.0.1257, 3D Systems GmbH, Moerfelden-Walldorf, Germany) and a biocompatible resin certified for the printing of surgical guides (SG, NextDent B. V., Soesterberg, The Netherlands). The post-processing was performed according to the manufacturer’s instructions with ethanol > 90% in an ultrasonic bath (Finevo Cleaning, sirius ceramics, Frankfurt, Germany). After light-curing for ten minutes at 60 ° Celsius (LC-3DPrint Box, 3D Systems GmbH, Moerfelden-Walldorf, Germany), metal sleeves were fixed into the templates: for the fully guided implant insertion a t-sleeve measuring 5.0 × 5.0 mm and for the pilot-drill guided implant insertion a t-sleeve measuring 2.2 × 6.0 mm (both Straumann Deutschland, Freiburg, Germany). A check of the fitting accuracy of the templates on the corresponding model was performed.

**Table 1:** Distribution of fully and pilot-drill guided implant insertion depending on the number of the model

Model	Region	Implant mode
Even number	36 (left)	fully guided
	46 (right)	pilot-drill guided
Uneven number	36 (left)	pilot-drill guided
	46 (right)	fully guided

**Fig. 2:**
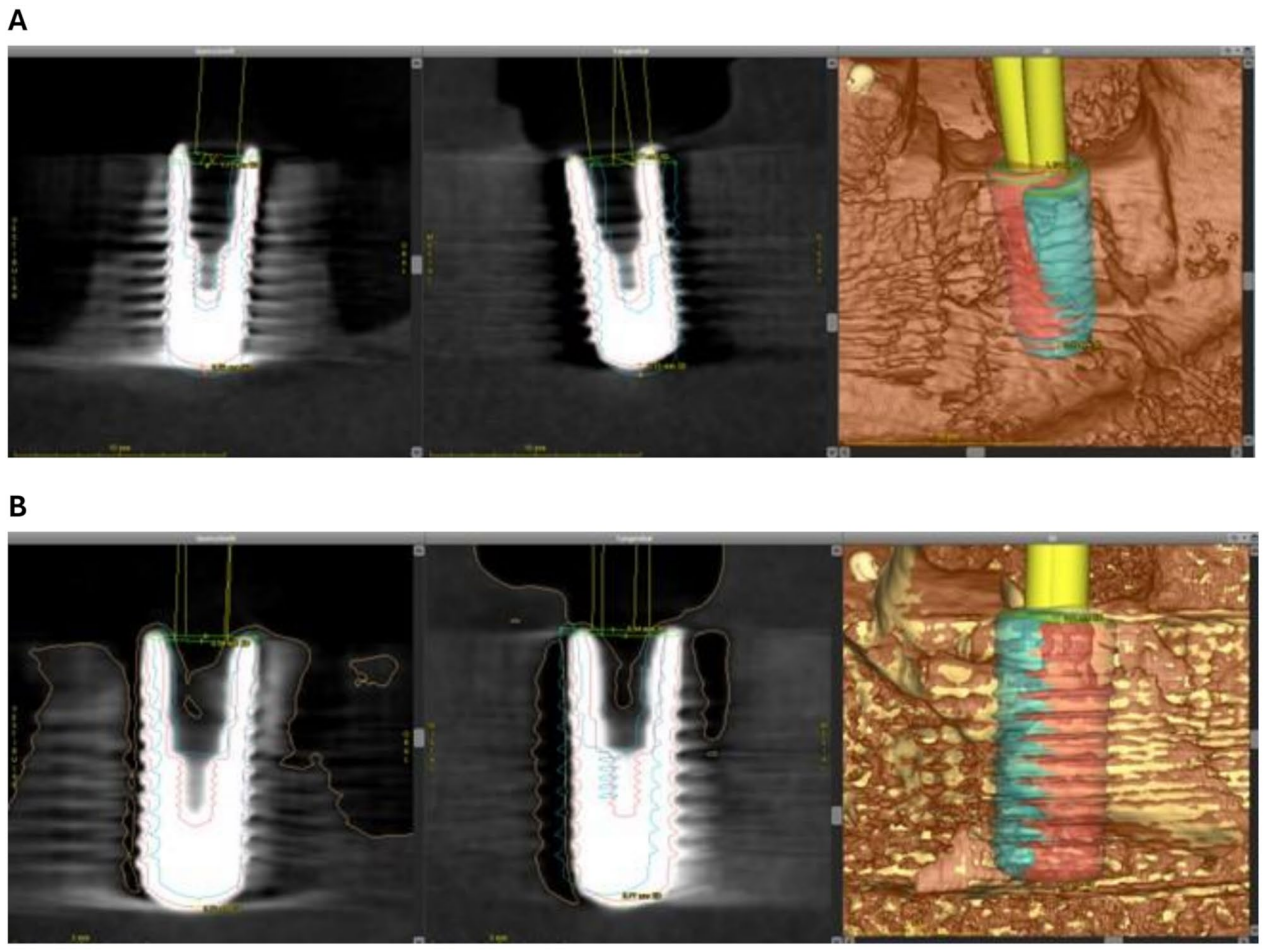
Three-dimensional superimposition of the pre-operatively planned position (blue) and the actual position (red) of the implant. (**A**) In this case, a distinctive distal deviation of the real achieved implant position in region 36 is visible. (**B**) In region 46, only a slight deviation to the distal direction is noticeable

Due to increased hygiene standards during the COVID-19 pandemic, the course had to be conducted in small groups including four participants. Prior to the beginning of the course, the participants were asked to complete a questionnaire comprising demographic data and a self-assessment of the participants’ knowledge of implant dentistry. Subsequently, an online presentation was given on the implant system and the surgical procedures. Next, the participants were planning the implant positions in the above-mentioned model situation in regions 36 and 46 using the coDiagnostiX™ software as an exercise. After completion of the theoretical part, the participants were asked to prepare the implant cavities in regions 36 and 46 in the models according to the manufacturer’s instructions. The implant cavities were prepared without irrigation using a surgical tray for guided implant insertion (Straumann Deutschland, Freiburg, Germany) and a surgical unit (Surgic-XT, NSK Europe, Eschborn, Germany). Whereas the pilot-drill guide was used once, the full guide was used for seven steps of which six required the application of the drill handle. Two identical dummy implants based on the Bone Level Regular Cross Fit (Straumann Deutschland, Freiburg, Germany) with a diameter of 4.1 mm and a length of 10 mm were inserted in the models. Next, the insertion abutments were removed. The time required from the initial insertion of the guide until end of the implant insertion at the crestal bone level was recorded. After inserting both implants, the participants were asked to complete the second part of the questionnaire containing the evaluation of the course and suggestions for potential improvement.

Subsequently, the mandibular models with the inserted implants were scanned by CBCT using the same parameters as pre-operatively. Next, the mismatch between the planned and the achieved implant position in oro-buccal, mesio-distal and cranio-caudal direction was evaluated using the i-dixel web (Version 2,16,1, J. Morita Corporation, Osaka, Japan). Furthermore, the axial deviation and the mismatch between the longitudinal axis of the sleeve and the implant were assessed in oro-buccal and mesio-distal direction. Additionally, the three-dimensional deviation between the planned and the achieved implant position were measured using the “treatment evaluation” tool of the coDiagnostiX™ software. Therefore, the pre- and the post-operative CBCT scans were matched by defining three congruent areas of each scan. Then, a superimposition of both scans was suggested by the software and manually adjusted by the examiner if necessary. Finally, the three-dimensional mismatch between planned and achieved implant position was calculated by the software (Fig. 2). To enable a determination of the direction of the mismatch, algebraic signs were applied: when the mismatch was in oral or mesial direction, a negative sign was used; when the mismatch was in buccal or distal direction, a positive sign was used. All values were captured in an MS Excel^®^ chart (Microsoft Inc., Redmond, Washington, USA).

### Statistical analysis

The statistical analysis was realized in co-operation with the Institute for Clinical Epidemiology and Applied Biometry, University Hospital Tübingen, Eberhard Karls University Tübingen, Germany by using the R Core Team (2021 R: A language and environment for statistical computing. R Foundation for Statistical Computing, Vienna, Austria). The mean values and their corresponding standard deviations were calculated. The predictability of the dependent variable from the predictor variables was estimated applying two-part generalized estimation equations (GEE) with zero inflation taking the dependency of observations due to being nested in students as well as an excess number of zeros into account. The comparison between the implant modes was analyzed using the Wilcoxon signed rank test for univariate group comparisons. The influence of sex, handedness, and a potential education prior to studying dentistry on the accuracy of the achieved implant position was analyzed using the t-Test (Welch’s test). The level of statistical significance was set up at α = 0.05.

## Results

### Demographic data

A total of 51 participants (35 female, 16 male) completed the course and inserted 102 implants. The mean age of the students was 27.3 years ranging from 22.8 to 35.2 years. The majority of the participants stated to be right-handed (44 participants), only seven stated to be left-handed. More than half of the participants had completed another education before studying dentistry. Detailed demographic data is depicted in Table 2.

**Table 2:** Demographical data of the participants

parameter	group	dental students
age	< 25 years	14
	> 25 years	37
gender	female	35
	male	16
handedness	left	7
	right	44
professional training before dental school	no	25
	yes	26
	dental technician	20
	dental assistent	5
	dental assistent/technician	1

### Evaluation models for the predictability of the dependent variables

For the average distribution of the values, a linear regression model was used. For the right skewed variables, a gamma regression model was applied. Thus, a prediction of the influence of a certain variable under the assumption that all other variables have a mean value is possible. The detailed values are shown in Table 3.

**Table 3:** Regression model

variable	implant mode	implant position	sex	handedness	previous education	regression model
absolute value mesio-distal mismatch						
estimates	0.48	1.26	0.98	1.11	0.82	gamma
CI	0.36–0.65	0.94–1.69	0.73–1.31	0.78–1.59	0.29–2.35	
*p*-value	**< 0.01**	0.12	0.87	0.56	0.72	
absolute value bucco-lingual mismatch						
estimates	0.45	0.94	0.81	0.84	2.49	gamma
CI	0.34–0.59	0.72–1.23	0.58–1.12	0.57–1.26	0.78–7.94	
*p*-value	**< 0.01**	0.63	0.20	0.40	0.12	
absolute value mesio-distal deviation						
estimates	0.45	1.66	0.98	0.65	0.45	gamma
CI	0.35–0.58	1.29–2.15	0.68–1.41	0.42–1.02	0.12–1.63	
*p*-value	**< 0.01**	**< 0.01**	0.92	0.06	0.22	
absolute value bucco-lingual deviation						
estimates	0.44	0.98	0.75	1.29	0.33	gamma
CI	0.32–0.59	0.73–1.32	0.55–1.03	0.88–1.89	0.11–1.00	
*p*-value	**< 0.01**	0.92	0.08	0.19	**< 0.05**	
mesio-distal mismatch at implant base						
estimates	0.18	-0.09	-0.13	0.06	-0.16	linear
CI	0.01–0.34	-0.25–0.08	-0.29–0.03	-0.14–0.25	-0.73–0.42	
*p*-value	**0.03**	0.30	0.11	0.58	0.60	
bucco-lingual mismatch at implant base						
estimates	-0.03	-0.07	-0.06	-0.18	-0.05	linear
CI	-0.22–0.17	-0.26–0.13	-0.28–0.15	-0.44–0.09	-0.82–0.72	
*p*-value	0.78	0.51	0.57	0.19	0.90	
apical mismatch at implant base						
estimates	0.33	-0.06	-0.06	0.13	0.20	linear
CI	0.14–0.53	-0.26–0.13	-0.29–0.16	-0.15–0.40	-0.61–1.01	
*p*-value	**< 0.01**	0.54	0.58	0.37	0.63	
3D mismatch at implant base						
estimates	0.74	1.10	1.12	0.94	1.19	gamma
CI	0.65–0.84	0.97–1.25	0.94–1.33	0.76–1.17	0.64–2.22	
*p*-value	**< 0.01**	0.13	0.22	0.60	0.58	
mesio-distal mismatch at implant tip						
estimates	0.02	-0.63	-0.22	0.30	-0.16	linear
CI	-0.26–0.31	-0.92–-0.34	-0.51–0.07	-0.06–0.66	-1.21–0.89	
*p*-value	0.87	**< 0.01**	0.14	0.11	0.76	
bucco-lingual mismatch at implant tip						
estimates	-0.36	-0.03	-0.23	-0.25	-0.61	linear
CI	-0.59–-0.13	-0.27–0.20	-0.52–0.07	-0.62–0.12	-1.69–0.46	
*p*-value	**< 0.01**	0.78	0.14	0.18	0.26	
apical mismatch at implant tip						
estimates	0.30	-0.05	-0.08	0.12	0.20	linear
CI	0.11–0.50	-0.25–0.14	-0.31–0.15	-0.16–0.40	-0.62–1.01	
*p*-value	**< 0.01**	0.60	0.50	0.40	0.63	
3D mismatch at implant tip						
estimates	0.75	1.39	1.12	0.95	1.41	gamma
CI	0.65–0.87	1.20–1.60	0.93–1.34	0.75–1.19	0.73–2.76	
*p*-value	**< 0.01**	**< 0.01**	0.25	0.65	0.31	
3D angle deviation						
estimates	0.48	1.42	0.95	1.01	1.64	gamma
CI	0.38–0.59	1.15–1.76	0.74–1.22	0.74–1.37	0.67–3.99	
*p*-value	**< 0.01**	**< 0.01**	0.67	0.95	0.28	
required time						
estimates	1.58	1.13	0.96	0.85	0.87	gamma
CI	1.44–1.75	1.02–1.24	0.78–1.19	0.66–1.11	0.40–1.86	
*p*-value	**< 0.01**	**< 0.05**	0.74	0.24	0.71	

Analysis of the dependent variables regarding implant mode, implant position, sex, handedness and a potential previous education. The estimates, their confidence intervals (CI) and the *p*-values are depicted. Statistically significant *p*-values are marked in bold font

### Deviation and mismatch between sleeve and inserted implant

When analyzing the deviation and the mismatch between the guiding sleeve and the inserted implant, the mean value for both parameters was smaller in the full-guided group. All differences between the mean values reached a statistical significance (*p* < 0.01). The deviation and mismatch in both groups pointed in the buccal and distal direction. The detailed data is shown in Table 4.

**Table 4:** Comparison of the accuracy for the implant insertion using the pilot-guided and the full-guided mode

parameter	group	mean value	± SD	mean difference	*p*-value	Wilcoxon paired RC
Mesio-distal mismatch implant/sleeve	Pilot-guided	0.38	0.28	0.19	**< 0.01**	0.61
(absolute value) in mm	Full-Guided	0.19	0.13			
Bucco-lingual mismatch implant/sleeve	Pilot-guided	0.44	0.26	0.25	**< 0.01**	0.82
(absolute value) in mm	Full-Guided	0.19	0.15			
Mesio-distal deviation implant/sleeve	Pilot-guided	7.22	4.45	3.69	**< 0.01**	0.66
(absolute value) in degrees	Full-Guided	3.53	2.84			
Bucco-lingual deviation implant/sleeve	Pilot-guided	8.63	5.63	4.81	**< 0.01**	0.70
(absolute value) in degrees	Full-Guided	3.82	3.12			
Mesio-distal mismatch at implant base	Pilot-guided	-0.72	0.45	-0.18	**< 0.05**	-0.39
in mm	Full-Guided	-0.55	0.30			
Bucco-lingual mismatch at implant	Pilot-guided	0.15	0.57	0.03	0.81	0.04
base in mm	Full-Guided	0.12	0.37			
Vertical mismatch at implant base	Pilot-guided	-0.55	0.43	-0.33	**< 0.01**	-0.52
in mm	Full-Guided	-0.22	0.53			
Cumulated mismatch at implant base	Pilot-guided	1.17	0.41	0.29	**< 0.01**	0.65
in mm	Full-Guided	0.88	0.31			
Mesio-distal mismatch at implant tip	Pilot-guided	-0.75	0.90	-0.01	0.76	-0.05
in mm	Full-Guided	-0.74	0.59			
Bucco-lingual mismatch at implant tip	Pilot-guided	0.47	0.73	0.36	**< 0.01**	0.55
in mm	Full-Guided	0.11	0.52			
Vertical mismatch at implant tip	Pilot-guided	-0.51	0.43	-0.30	**< 0.01**	-0.48
in mm	Full-Guided	-0.21	0.53			
Cumulated mismatch at implant tip	Pilot-guided	1.48	0.60	0.35	**< 0.01**	0.44
in mm	Full-Guided	1.13	0.45			
Three-dimensional angle	Pilot-guided	4.51	2.20	2.27	**< 0.01**	0.78
in degrees	Full-Guided	2.24	1.38			

Mean values, standard deviation (SD), the mean difference, *p*-value and the Wilcoxon rank biserial correlation coefficient (Wilcoxon paired rc) are depicted. The *p*-values resulted from a pair-wise comparison. *P*-Values for statistically significant differences are marked in bold font. Effect of the matched-pairs rank biserial correlation coefficient: 0–30 = small, 30–50 = medium, > 50 large

### Differences between virtually planned and really inserted implant

When comparing the virtually planned implant position with the really achieved implant position, it could be observed that the fully guided implant insertion led to higher accuracy compared to the pilot-drill guided. For the bucco-lingual mismatch at the implant base and the mesio-distal mismatch at the implant tip, the differences were neglectable. For all other values, the differences measured between fully guided and pilot-drill guided implant insertion reached a statistical significance. The highest mean difference between both methods was obtained for the three-dimensional angle of the implant axis with 2.27 degrees. The detailed values are depicted in Table 4.

### Individual factors

When considering the individual factors, e.g. sex, handedness and a potential previous education, a tendency could not be found. Only three values reached a statistically significant difference between the analyzed groups: For the bucco-lingual axial deviation female and male participants showed a deviation to the oral direction. However, this deviation was statistically significantly higher for the male students (-6.58 ± 3.35 degrees vs. -4.39 ± 3.97 degrees, *p* < 0.05). Furthermore, left- and right-handed participants revealed a mismatch to the mesial direction at the implant tip. When analyzing the differences between left- and right-handed students, a statistically significant higher mismatch was obvious in the left-handed group (-1.02 ± 0.33 mm vs. -0.70 ± 0.47 mm, *p* < 0.05). The last value showing a statistically significant difference between two groups was the mesio-distal mismatch between the sleeve and the implant base when comparing participants without a previous education (-0.28 ± 0.18 mm) to students with a previous education (-0.15 ± 0.23 mm, *p* < 0.05). All other analyzed values indicated no statistically significant differences.

### Required time for planning of the implant position and implant insertion

The time required for the planning procedure using the software was 10:27 ± 3:20 min for each implant. The same planning procedure was applied for both guiding modes. Thus, no statistical analysis was performed.

The time recorded for the pilot-drill guided implant insertion was 9:35 ± 3:58 min compared to 15:22 ± 5:22 min for the full-guided implant insertion. This difference reached a statistical significance (*p* < 0.01). The detailed data is shown in Table 5. No statistically significant differences were obvious regarding the sex, handedness or whether the participants had completed another education prior to the study of dentistry.

**Table 5:** Comparison of the time required for implant insertion for the two groups

parameter	group	mean value	± SD	minium	maximum	difference	*p*-value	Wilcoxon paired rc	
required time	Pilot-guided	09:35	03:58	04:20	21:01	05:47	**< 0.01**	-0.96	
in minutes	Full-Guided	15:22	05:22	06:40	34:00				

Mean values, standard deviation (SD), minima and maxima, the difference, *p*-value and the Wilcoxon rank biserial correlation coefficient (Wilcoxon paired rc) are depicted. The *p*-value resulted from a pair-wise comparison. *P*-Values for statistically significant differences are marked in bold font. Effect of the matched-pairs rank biserial correlation coefficient: 0–30 = small, 30–50 = medium, > 50 large

### Evaluation of the questionnaires

The evaluation of the questionnaires revealed that the majority of the students had either none or only in a theoretical way contact with implant dentistry. When self-assessing the knowledge of dental implants most of the students stated that they have little or sketchy previous knowledge. The majority of the participants was highly interested in the surgical and prosthetic part of implant dentistry.

After the completion of the workshop, most of the students stated that the course was rather or absolutely meeting their expectations. Considering the answers before and after the course, the interest in the prosthetic and surgical parts of implant dentistry could be aroused. Furthermore, the participants required a more profound education in surgical procedures, planning of dental implants and incision techniques. The detailed results of the questionnaires are presented in Table 6.

**Table 6:** Results of the questionnaire

Statements prior participation in the course							
What was your previous contact with oral implants?	none	theoretical	practical	theoretical/practical	other	not stated	
	14	32	1	0	7	0	
How would assess your knowledge in oral implantology?	little	sketchy	sufficient	extensive	not stated		
	28	20	3	0	0		
Are you interested in the surgical part of oral implantology?	no	rather no	rather yes	absolutely	not stated		
	0	1	17	33	0		
Are you interested in the prosthetic part of oral implantology?	no	rather no	rather yes	absolutely	not stated		
	0	1	19	31	0		
Would you get a dental implant if necessary?	no	rather no	rather yes	absolutely	not stated		
	0	0	20	30	1		
Statements after participation in the course							
Have your expectations considering the course been fulfilled?	no	rather no	rather yes	absolutely	not stated		
	0	0	8	42	1		
How do you evaluate your education in oral implantology?	poor	sketchy	sound	extensive	not stated		
	9	24	9	7	2		
Are you interested in the surgical part of oral implantology?	no	rather no	rather yes	absolutely	not stated		
	0	1	11	38	1		
Are you interested in the prosthetic part of oral implantology?	no	rather no	rather yes	absolutely	not stated		
	0	1	13	36	1		
Would you get a dental implant if necessary?	no	rather no	rather yes	absolutely	not stated		
	0	0	21	29	1		
Which topics would you be interested in considering oral implantology?	planning	incision techniques	surgical procedures	suture techniques	impressions	prosthetics	not stated
(multiple selections allowed)	45	34	46	26	22	31	1

The previous knowledge and experiences as well as the interests of the participants considering oral implantology are assessed. After completing the course, the participants were additionally asked to evaluate the course and the education in oral implantology

## Discussion

### Comparison of accuracy of pilot-drill guided and full-guided implant insertion

When comparing the values for the accuracy of the implant insertion using the fully guided mode all values were higher when compared to the pilot-drill guided. This is in line with the findings of previous laboratory studies [5, 6, 9, 25]. The closer guidance provided by the fully guided implant insertion might be beneficial for inexperienced surgeons as extended mistakes are unlikely when the manufacturer’s protocol is followed. When beginning with implant dentistry, the safety for inexperienced surgeons might thus increase. However, even with fully guided implant insertion a residual error has to be considered. First, there might be a mismatch when superimposing the surface scan over the data set obtained by cone beam computed tomography. Furthermore, the template itself must have a clearance fit in order to be positioned on the model. In the current study, the offset was 0.2 mm for the templates. Additionally, the guiding sleeve must have tolerance in order to allow a smooth gliding of the implant bur. This tolerance was reported with 60 μm for the sleeve and might cause a deviation of 200 μm on the tip of the bur depending on its lengths [26]. However, all these factors were leveled as the same procedure and materials were used except for the guiding mode. When using the fully guided implant insertion, the mean mismatch was less than 1 mm, and the mean angular deviation was 2.24 degrees. Those findings are comparable to the findings of Ebeling et al. who were comparing the deviation between undergraduates and implantologists using the same system [22]. The slightly higher deviations in their study compared to the current examination might be due to the use of phantom heads. In our study, the models were not fixed in a phantom head so that the participants were able to have an overview of and access to the model from all directions enabling enhanced visual control and possibility for corrections.

For the vertical mismatch, there was a tendency that the implants were not inserted deep enough. One potential reason might be the consistency of the resin that the models were made from. As the drilling experience was rather rigid, a higher torque was needed to insert the implants. Another reason might be that the preparation of the cavities was performed without irrigation. Thus, debris might not have been fully cleared from the implant cavity and has prevented the implant from being inserted to the vertical stop position. Considering the vertical mismatch, no value either for the fully guided nor for the pilot-drill guided implant insertion was more than 2 mm. Certainly, this might be important as a regular safety margin is 2 mm. Thus, an injury of the adjacent anatomical structures e.g. the inferior alveolar nerve, would be unlikely when using a template. When comparing the results of fully guided and pilot-drill guided implant insertion, it can be stated that both methods provide sufficient accuracy within the safety margin of 2 mm.

### Influence of individual factors on the accuracy

The influence of the individual factors is a bit controversial as only single parameters have reached statistical significance. This is in accordance with previous examinations having analyzed undergraduate students [7–9]. In contrast, van de Wiele et al. found a higher accuracy in right-handed surgeons for implant placement on the right site and for left-handed implant placement on the left site in the inexperienced group [27]. One reason could be that in the present examination tooth-borne templates were used in a laboratory set-up whereas Van de Wiele et al. used mucosa-borne templates in a clinical set-up. In different studies, tooth-borne templates showed a higher accuracy when compared to mucosa-borne templates [28, 29]. This might be due to the higher resilience of the mucosa compared to natural teeth or bone. Furthermore, the positioning of the templates is more reliable when teeth are available for fixation. Another potential reason for not finding statistically significant differences considering the individual factors in the present examination might be that the examination used a laboratory set-up. Thus, the participants were able to overview the surgical site without restrictions and the models were not fixed so that they could be positioned according to the participant’s need.

### Time required for implant insertion

In the current examination, the participants needed statistically significantly more time to insert the implants fully guided compared to the pilot-drill guided implant insertion. This might be explained by the fact that the pilot-drill guided implant insertion requires only once the application of the drill into the sleeve, whereas the full-guided implant insertion requires this procedure seven times potentially leading to an extended time for implant cavity preparation and implant insertion. The literature reporting the time required for implant insertion is contradictory [7, 9]. In one study, the participants needed statistical significantly less time for fully guided implant insertion [7] while in another examination the fully guided implant insertion took longer even though the difference reached no statistical significance [9]. In a recent study, mandibular models were mounted in a phantom head and fully guided implant insertion was performed with two different guides, the time required for the procedure was comparable to time recorded in the current study 16:26 ± 5:52 min versus 15:22 ± 05:22 min [30]. In general, the participants in the current study required more time for implant insertion in general and statistically significantly more for fully guided implant insertion. Another potential reason for the longer time required for fully guided implant placement in the current study could be the implant system. In the studies mentioned, a surgical tray using drill sleeves already placed on the respective burr was used. The currently applied guided surgery tray uses a drill handle that has to be placed into the sleeve in the template. Especially when the gap is narrow, placing the drill handle in the template sleeve correctly without twisting can be challenging. For the pilot-drill guided implant insertion, the pilot drill has to be placed into the template sleeve without the drill handle. As the participants were not familiar with the guided surgery tray, the correct positioning of the drill handle might be the reason for the longer time observed in the current study.

### Questionnaire

The completion of the questionnaire was divided into one part prior to and one part after participation in the hands-on course. Surprisingly, a majority had only theoretical knowledge in implant dentistry although 26 of the participants had a dentistry-related professional education prior to studying dentistry. This fact is specific to our Dental School as applicants with a completed education as dental assistant or dental technician get a bonus in the university election process for the allocation of places in higher education. In a recent study, a comparable ratio of students with completed dentistry-related education could be observed [30]. Initially, most of the participants stated to have insufficient knowledge in implant dentistry which is in line with a previous examination at a German Dental School [9]. A potential explanation might be the hygiene restriction during the COVID-19 pandemic. In this term, practical education was reduced, and elective surgery had to be postponed which reduced the possibility for the students to obtain practical experience considering implant dentistry. After the course, the interest in implant dentistry could be increased according to the questionnaires. These results are in line with findings from other countries where the surveys were yielding an extended interest in implant dentistry [17, 31]. However, combinations of theory and practical training are time-consuming and require staff. According to a survey in the United Kingdom, major limiting factors in teaching implant dentistry are limited time and funding and insurance liability [32]. This is in line with the situation in the presented examination. As funding is the most urgent limiting factor, there is the necessity of support from implant providers. However, this fact always raises the concern of a lack of independence in dental education. A potential way to overcome this concern might be partnerships with multiple different implant providers in order to avoid a bias toward one specific system [32].

### Teaching method

The teaching method applied in the current study was a three-step procedure. First, the theoretical lesson was performed online. The digital planning of the implant position was the second step. Subsequently, the practical training followed. It would have been advantageous to integrate blended learning into the course e.g. the digital planning of the implant position as an online course in advance to the practical part as it has shown beneficial long-time results [33]. However, due to the license management of the planning software, it was not possible to perform the planning online. Thus, the planning part was done on-site on licensed workstations. Furthermore, a long-term evaluation was not part of the current study. One drawback of hands-on teaching is that it is cost-intensive and time-consuming. When totalizing the material costs per participant, it is adding up to 150 € not included the working time of the staff. This fact leads to another disadvantage: The application of the Peyton-4-step approach was not possible due to limited resources. In a recent study of our department, promising results could be shown for the use of this method for the teaching of surgical sutures [34]. As the 4-step approach contains demonstration–deconstruction–comprehension, and performance by the learner the procedure of the implant insertion by the instructor would have been necessarily performed multiple times during the course. However, due to the limited resources considering the material, this was not possible. In future courses, a video sequence of the instructor performing the implant insertion would be advantageous.

### Limitations

As the examination was in a laboratory set-up, the results have to be interpreted with caution. The implants were inserted into mandibular models with a gingiva mask in the region of implant placement that were not mounted into a phantom head. Constricting factors being present in a clinical situation, e.g. bleeding, soft tissue, limited mouth opening, or saliva were absent. The participants were able to have a clear view and access from all directions to the model. Thus, the implant insertion in the presented study might be considered as easier compared to a clinical situation [3]. Furthermore, it can be assumed that the time required for implant insertion is different from a clinical situation. The mandibular models are another limiting factor. The resin is not imitating the structure of natural bone [22]. Furthermore, the resin debris cannot be removed by irrigation so that it might remain in the implant cavity potentially leading to deviations, certainly vertical, when inserting the implant. On the other hand, laboratory training is an important step before performing surgery in real patients. Offering the opportunity for the instructors to give direct feedback to the participants considering their accuracy provides a quality-controlled training which has been reported to have a positive effect on the patients’ safety [23]. Another factor potentially influencing the results is the fact that, due to hygiene restrictions caused by the COVID-19 pandemic, the course was only possible in small groups of four students. Thus, closer support and supervision was assured compared to larger groups. This close supervision might lead to more accurate results as errors could be identified early and corrected compared to the hands-on courses in larger groups. However, teaching in small groups is favored but has higher staff and time requirements. Therefore, considering the limited resources available in dental education, training in small groups is preferred but a return to courses in larger groups might be unavoidable.

## Conclusion

Within the limits of the study, the results showed that the application of plotted templates allows a more correct and reproducible insertion of dental implants for undergraduates in a laboratory set-up. The hypothesis, that fully guided implant insertion is more accurate compared to pilot-drill guided could be confirmed. The second hypothesis, that the fully guided implant insertion requires more time has been confirmed, likewise. Based on the evaluation of the questionnaires, there is much interest in theory and practical training in implant dentistry among undergraduate students.

## Data Availability

The datasets supporting the conclusions of this article are available. Availability of data and materials by the corresponding author: Matthias.Schulz@med.uni-tuebingen.de.

## Abbreviations

CBCT: Cone beam computed tomography
COVID: 19–Coronavirus SARS–CoV–2
DICOM: Digital imaging and communications in medicine
kV: Kilo Volt
mA: Milli Ampere
mm: Millimeter
µm: Micrometer

## References

[1] Matta R-E, Bergauer B, Adler W, Wichmann M, Nickenig H-J. The impact of the fabrication method on the three-dimensional accuracy of an implant surgery template. J cranio-maxillo-facial Surgery: Official Publication Eur Association Cranio-Maxillo-Facial Surg. 2017;45:804–8. 10.1016/j.jcms.2017.02.015 10.1016/j.jcms.2017.02.015 28363503

[2] Gargallo-Albiol J, Barootchi S, Salomó-Coll O, Wang H-L. Advantages and disadvantages of implant navigation surgery. A systematic review. Annals Anat = Anatomischer Anzeiger: Official Organ Anatomische Gesellschaft. 2019;225:1–10. 10.1016/j.aanat.2019.04.005 10.1016/j.aanat.2019.04.005 31063802

[3] H-J Nickenig N, Wichmann M, Hamel J, Schlegel K, Eitner S. Evaluation of the difference in accuracy between implant placement by virtual planning data and surgical guide templates versus the conventional free-hand method - a combined in vivo - in vitro technique using cone-beam CT (Part II). J cranio-maxillo-facial Surgery: Official Publication Eur Association Cranio-Maxillo-Facial Surg. 2010;38:488–93. 10.1016/j.jcms.2009.10.023 10.1016/j.jcms.2009.10.023 19939691

[4] Scherer U, Stoetzer M, Ruecker M, Gellrich N-C, See C. Template-guided vs. non-guided drilling in site preparation of dental implants. Clin Oral Invest. 2015;19:1339–46. 10.1007/s00784-014-1346-7 10.1007/s00784-014-1346-7 25354488

[5] Vermeulen J. The accuracy of implant placement by experienced surgeons: guided vs freehand approach in a simulated plastic model. Int J Oral Maxillofac Implants. 2017;32:617–24. 10.11607/jomi.5065 27741330 10.11607/jomi.5065

[6] Ketabi A-R, Kastner E, Brenner M, Lauer H-C, Schulz MC. Implant insertion using an orientation template and a full-guiding template - A prospective model analysis in a cohort of dentists participating in an implantology curriculum. Annals Anat = Anatomischer Anzeiger: Official Organ Anatomische Gesellschaft. 2021;236:151716. 10.1016/j.aanat.2021.151716 10.1016/j.aanat.2021.151716 33675946

[7] Schulz MC, Hofmann F, Range U, Lauer G, Haim D. Pilot-drill guided vs. full-guided implant insertion in artificial mandibles-a prospective laboratory study in fifth-year dental students. Int J Implant Dentistry. 2019;5:23. 10.1186/s40729-019-0176-4 10.1186/s40729-019-0176-4 PMC6593025 31240421

[8] Schulz MC, Rittmann L, Range U, Lauer G, Haim D. The use of orientation templates and free-hand implant insertion in artificial mandibles-an experimental laboratory examination in fifth-year dental students. Dentistry J. 2018. 10.3390/dj6030043 10.3390/dj6030043 PMC6162789 30200450

[9] Schulz MC, Tokarski M, Jacoby J, Naros A, Weise C, Tausche E, et al. Accuracy of full-guided vs. pilot-guided implant insertion - a prospective laboratory study in fifth-year dental students. Annals Anat = Anatomischer Anzeiger: Official Organ Anatomische Gesellschaft. 2023;248:152082. 10.1016/j.aanat.2023.152082 10.1016/j.aanat.2023.152082 36913983

[10] Wegmüller L, Halbeisen F, Sharma N, Kühl S, Thieringer FM. Consumer vs. High-End 3D printers for guided implant Surgery-An in vitro accuracy assessment study of different 3D printing technologies. J Clin Med. 2021. 10.3390/jcm10214894 34768413 10.3390/jcm10214894 PMC8584299

[11] Kernen F, Kramer J, Wanner L, Wismeijer D, Nelson K, Flügge T. A review of virtual planning software for guided implant surgery - data import and visualization, drill guide design and manufacturing. BMC Oral Health. 2020;20:251. 10.1186/s12903-020-01208-1 32912273 10.1186/s12903-020-01208-1 PMC7488021

[12] Wang W, Zhuang M, Li S, Shen Y, Lan R, Wu Y, Wang F. Exploring training dental implant placement using static or dynamic devices among dental students. Eur J Dent Education: Official J Association Dent Educ Europe. 2023;27:438–48. 10.1111/eje.12825 10.1111/eje.12825 35579548

[13] Cushen SE, Turkyilmaz I. Impact of operator experience on the accuracy of implant placement with stereolithographic surgical templates: an in vitro study. J Prosthet Dent. 2013;109:248–54. 10.1016/S0022-3913(13)60053-0 23566606 10.1016/S0022-3913(13)60053-0

[14] Atay E, Hey J, Beuer F, Böse MWH, Schweyen R. Evaluation of the accuracy of fully guided implant placement by undergraduate students and postgraduate dentists: a comparative prospective clinical study. Int J Implant Dentistry. 2024;10:6. 10.1186/s40729-024-00526-1 10.1186/s40729-024-00526-1 PMC10850045 38324168

[15] Sánchez-Garcés M-A, Berástegui-Jimeno E, Gay-Escoda C. Knowledge, aptitudes, and preferences in implant dentistry teaching/training among undergraduate dental students at the university of Barcelona. Med Oral Patologia Oral Y Cir Bucal. 2017;22:e484–90. 10.4317/medoral.21741 PMC5549522 28578375

[16] Sharma A, Shrestha B, Chaudhari BK, Suwal P, Singh RK, Niraula SR, Parajuli PK. Preferred source and perceived need of more information about dental implants by the undergraduate dental students of nepal: all Nepal survey. Int J Dent. 2018;2018:6794682. 10.1155/2018/6794682 29713346 10.1155/2018/6794682 PMC5866872

[17] Chaturvedi S, Elmahdi AE, Abdelmonem AM, Haralur SB, Alqahtani NM, Suleman G, et al. Predoctoral dental implant education techniques-students’ perception and attitude. J Dent Educ. 2021;85:392–400. 10.1002/jdd.12453 33067837 10.1002/jdd.12453

[18] Shetty SR, Murray CA, Al Kawas S, Jaser S, Al-Rawi N, Talaat W, et al. Impact of fully guided implant planning software training on the knowledge acquisition and satisfaction of dental undergraduate students. Med Educ Online. 2023;28:2239453. 10.1080/10872981.2023.2239453 37490557 10.1080/10872981.2023.2239453 PMC10392243

[19] Schweyen R, Al-Nawas B, Arnold C, Hey J. A cross-sectional survey of attitudes towards education in implant dentistry in the undergraduate dental curriculum. Int J Implant Dentistry. 2020;6:26. 10.1186/s40729-020-00224-8 10.1186/s40729-020-00224-8 PMC7340723 32638176

[20] Chin JS, Lynch CD, Rees J, Locke M, Thomas MBM, Addy LD. Teaching of implant dentistry in undergraduate dental schools in the UK and Ireland. British Dental Journal. 2018. 10.1038/sj.bdj.2018.867 10.1038/sj.bdj.2018.867 30337728

[21] Sharma A, Chaudhari BK, Shrestha B, Suwal P, Parajuli PK, Singh RK, Niraula SR. Knowledge and perception about dental implants among undergraduate dental students. BDJ Open. 2019;5:1. 10.1038/s41405-018-0009-1 30886741 10.1038/s41405-018-0009-1 PMC6418164

[22] Ebeling M, Sakkas A, Schramm A, Wilde F, Scheurer M, Winter K, Pietzka S. Accuracy analysis of computer-assisted and guided dental implantology by comparing 3D planning data and actual implant placement in a mandibular training model: a monocentric comparison between dental students and trained implantologists. J Pers Med. 2023. 10.3390/jpm13071037 37511650 10.3390/jpm13071037 PMC10381824

[23] Kim NY, Stagnell S. Postgraduate education in dental implantology in the united kingdom: a review. Int J Implant Dentistry. 2018;4:8. 10.1186/s40729-017-0115-1 10.1186/s40729-017-0115-1 PMC5789118 29380084

[24] World Medical Association. Declaration of helsinki: ethical principles for medical research involving human subjects. JAMA. 2013;310:2191–4. 10.1001/jama.2013.281053 24141714 10.1001/jama.2013.281053

[25] Bilhan H, Arat S, Mumcu E, Geckili O, Sakar O. Precision of implant placement with stereolithographic templates: a pilot in vitro study. J Oral Implantol. 2012;38:569–74. 10.1563/AAID-JOI-D-10-00109 21126171 10.1563/AAID-JOI-D-10-00109

[26] Dreiseidler T, Tandon D, Ritter L, Neugebauer J, Mischkowski RA, Scheer M, Zoller JE. Accuracy of a newly developed open-source system for dental implant planning. Int J Oral Maxillofac Implants. 2012;27:128–37. 22299089

[27] van de Wiele G, Teughels W, Vercruyssen M, Coucke W, Temmerman A, Quirynen M. The accuracy of guided surgery via mucosa-supported stereolithographic surgical templates in the hands of surgeons with little experience. Clin Oral Implants Res. 2015;26:1489–94. 10.1111/clr.12494 25318961 10.1111/clr.12494

[28] Turbush SK, Turkyilmaz I. Accuracy of three different types of stereolithographic surgical guide in implant placement: an in vitro study. J Prosthet Dent. 2012;108:181–8. 10.1016/S0022-3913(12)60145-0 22944314 10.1016/S0022-3913(12)60145-0

[29] Shi Y, Wang J, Ma C, Shen J, Dong X, Lin D. A systematic review of the accuracy of digital surgical guides for dental implantation. Int J Implant Dentistry. 2023;9:38. 10.1186/s40729-023-00507-w 10.1186/s40729-023-00507-w PMC10597938 37875645

[30] Schulz MC, Krimmel M, Weismann C, Kaucher-Fernandez P, Lethaus B, Mann NK. Influence of two different printing methods on the accuracy of full-guided implant insertion - a laboratory study in undergraduate dental students. BDJ Open. 2025;11:6. 10.1038/s41405-025-00295-y 39865072 10.1038/s41405-025-00295-y PMC11770064

[31] Homma S, Sasaki H, Furuya Y, Ito T, Yajima Y. Current state of undergraduate education in oral implantology in Japan. Bull Tokyo Dent Coll. 2015;56:73–83. 10.2209/tdcpublication.56.73 26084995 10.2209/tdcpublication.56.73

[32] Hare A, Bird S, Wright S, Ucer C, Khan RS. Current undergraduate dental implantology teaching in UK. Dentistry J. 2022. 10.3390/dj10070127 10.3390/dj10070127 PMC9324480 35877401

[33] Blond N, Chaux A-G, Hascoët E, Lesclous P, Cloitre A. Blended learning compared to traditional learning for the acquisition of competencies in oral surgery by dental students: a randomized controlled trial. Eur J Dent Education: Official J Association Dent Educ Europe. 2024;28:943–54. 10.1111/eje.13030 10.1111/eje.13030 39083448

[34] Leitmann A, Reinert S, Weise H. Surgical suture course for dental students with the Peyton-4-step approach versus the PDCA cycle using video assisted self-monitoring. BMC Oral Health. 2020;20:36510.1186/s12903-020-01309-x 33380320 10.1186/s12903-020-01309-x PMC7772909

